# Pediatric Response to Second-Line Antiretroviral Therapy in South Africa

**DOI:** 10.1371/journal.pone.0049591

**Published:** 2012-11-20

**Authors:** Brian C. Zanoni, Henry Sunpath, Margaret E. Feeney

**Affiliations:** 1 The Ragon Institute of Massachusetts General Hospital, Massachusetts Institute of Technology, and Harvard, Charlestown, Massachusetts, United States of America; 2 Harvard Medical School, Boston, Massachusetts, United States of America; 3 Sinikithemba Clinic and Philani Program, McCord Hospital, Durban, South Africa; 4 Infectious Diseases Unit, Nelson Mandela School of Medicine, Durban, South Africa; 5 Division of Experimental Medicine, University of California San Francisco, San Francisco, California, United States of America; University of Pittsburgh, United States of America

## Abstract

**Background:**

With improved access to pediatric antiretroviral therapy (ART) in resource-limited settings, more children could experience first-line ART treatment failure.

**Methods:**

We performed a retrospective cohort analysis using electronic medical records from HIV-infected children who initiated ART at McCord Hospital's Sinikithemba Clinic in KwaZulu-Natal, South Africa, from August 2003 to December 2010. We analyzed all records from children who began second-line ART due to first-line treatment failure. We used logistic regression to compare viral outcomes in Protease Inhibitor (PI)-based versus Non-Nucleoside Reverse Transcriptase Inhibitor (NNRTI)-based second-line ART, controlling for time on first-line ART, sex, and whether HIV genotyping guided the regimen change.

**Results:**

Of the 880 children who initiated ART during this time period, 80 (9.1%) switched to second-line ART due to therapeutic failure of first-line ART after a median of 95 weeks (IQR 65–147 weeks). Eight (10%) of the failures received NNRTI-based second-line ART, all of whom failed a PI-based first-line regimen. Seventy (87.5%) received PI-based second-line ART, all of whom failed a NNRTI-based first-line regimen. Two children (2.5%) received non-standard dual therapy as second-line ART. Six months after switching ART regimens, the viral suppression rate was significantly higher in the PI group (82%) than in the NNRTI group (29%; p = 0.003). Forty-one children (51%) were tested for genotypic resistance prior to switching to second-line ART. There was no significant difference in six month viral suppression (p = 0.38) between children with and without genotype testing.

*Conclusion:* NNRTI-based second-line ART carries a high risk of virologic failure compared to PI-based second-line ART.

## Introduction

Since 2005, there has been a dramatic increase in ART access for HIV-infected children in sub-Saharan Africa [1], [2], [3]. However, the availability of adequate care and treatment programs remain limited [4] and most treatment programs in developing countries have a restricted formulary of antiretroviral medications, particularly for children. Resistance to first-line ART is an increasing problem [5], [6], [7], [8]. With the limited treatment options available, choosing the correct second-line therapy is critical [4], [9], [10], yet resistance testing is not available in most resource-limited settings. Increasing use of single-dose nevirapine (NVP) in Prevention of Mother to Child Transmission (PMTCT) programs could limit the effectiveness of non-nucleoside reverse transcriptase inhibitors (NNRTI) in younger children [11], [12]. Archived resistance mutations in the NVP-exposed infants could potentially limit both first- and second-line use of NNRTI in resource-limited settings [10], [13].

We performed a retrospective cohort study to evaluate the response to second-line ART in children in South Africa by comparing NNRTI-based second-line ART with PI-based second- line ART. In addition, we used existing resistance data to compare outcomes between children receiving standard second-line ART and those whose regimen change was guided by resistance testing.

## Materials and Methods

### Ethics Statement

The protocol was approved by McCord Hospital's Research Ethics Committee and the Partners Human Research Committee. All patients and their adult caregivers accessing care at McCord Hospital signed a written consent to have their medical information stored on an electronic medical record database used for clinical and research purposes.

### Study design

We performed a retrospective cohort study using electronic medical records from HIV-infected pediatric patients (≤18 years old) who initiated antiretroviral therapy at McCord Hospital's Sinikithemba Clinic in KwaZulu-Natal, South Africa, from August 2003 to December 2010. We analyzed all records from children who changed their regimen from first-line ART. We recorded clinical and demographic information at baseline prior to ART initiation and collected six monthly CD4, viral load, weight, and ALT, and hemoglobin to evaluate the response to second-line ART.

### Study population and standard of care

McCord Hospital is a semi-private, urban hospital providing care for a mostly Zulu-speaking population in Durban, South Africa. We followed patients in the study from the time they initiated ART until they died, transferred care to another facility, were lost to follow-up, or until the study end date of December 31, 2010. During the study period, children initiated ART when their HIV disease reached World Health Organization (WHO) stage 3 or 4 and/or their CD4 percentage was less than 20% in children younger than 18 months, or less than 15% in children older than 18 months, in accordance with South African National Treatment Guidelines [14]. Based on national guidelines in South Africa, children less than 3 years of age received a PI-based first-line treatment regimen comprised of lopinavir/ritonavir, stavudine, and lamivudine [14]. Children older than 3 years initiated an NNRTI-based treatment regimen comprised of efaverinez, stavudine, and lamivudine [14]. According to local guidelines, routine laboratory monitoring included baseline CD4 and six monthly CD4 and viral loads [14]. The South African National Treatment Guidelines define virologic failure as two consecutive viral loads greater than 1,000 copies/ml after six months of ART, despite adherence, with viral loads separated by three months [15]. Children failing an NNRTI-based regimen were changed to standard second-line ART of zidovudine (AZT), didanosine (DDI), and lopinavir/ritonavir. Children failing a PI-based regimen received AZT, DDI, and efaverinez.

### Resistance Testing

Resistance testing was performed from January 1, 2005 to August 15, 2006 for consecutive children <18 years with a viral load >1,000 copies/ml under a separate research protocol. Genotyping of plasma virus was performed using the TRUGENE® HIV-1 Genotyping Test on an OpenGene® DNA Sequencing System (Bayer HealthCare Diagnostics, Berkeley, CA) as directed by the manufacturer. Confirmatory HIV-1 RNA testing using the NucliSens EasyQ HIV-1 (bioMeriux diagnostics, Marcy l'Etoille, France) was performed for specimens with either initial results of <10,000 copies/mL or if viral RNA could not be amplified for sequencing. Substitutions at the following positions were considered major drug resistance mutations: for reverse transcriptase (RT), M41L, K65R, D67N, insertion 69, K70R, L74V, L100I, K103N, V106A/M, V108I, Q151M, Y181C, M184V, Y188C/L, G190A, L210W, T215Y/F, K219Q/E/N/R, P225H, and M230L; for protease (PR), D30N, V32I, L33F/I, M46I/L, I47V/A, G48V, I50V, V82A/T/F/S, I84V, and L90M. During this study period, clinicians used results of the resistance testing to guide choice in NRTI backbone; however, national guidelines dictated choice of PI or NNRTI.

### Data Collection

We evaluated medical records from patients aged ≤18 years old who changed ART regimens at McCord Hospital's Sinikithemba Clinic from August 2003 to December 2010. TrackCare Software was used to maintain electronic medical records. All records were cross referenced with paper charts. Collected data included age at ART initiation, gender, ART regimens, presence of tuberculosis (TB) and non-TB opportunistic infections, chronic diarrhea (longer than 14 days), baseline and six monthly laboratory results including absolute and CD4 percentage, viral load and hemoglobin. At the time of ART regimen change, we recorded whether or not the children had resistance testing performed and whether they had major resistance mutations. We also recorded the presence of chronic diarrhea and opportunistic infections based on documentation in the electronic medical record as well as review of paper records. If these conditions were not documented in the electronic medical record or paper charts, we reported them as absent.

### Statistical Analysis

We conducted statistical analyses using SAS statistical software (Release 9.2, Carey, NC). We first determined univariate associations between nine demographic and clinical covariates, which, based upon clinical observations and prior studies, were hypothesized as potentially important correlates of response to second-line ART. We then performed multivariate logistic regression controlling for age, sex, ART regimen, and presence of resistance testing.

## Results

Between August 2003 and December 2010, 880 children initiated ART at McCord Hospital's Sinikithemba Clinic. Of these children, 186 (21%) changed ART from their initial regimen prior to December 31, 2010. Of those, 80 (9.1%) were due to virologic failure and 106 (12%) were due to toxicity, intolerance, or a change in national guidelines. Risk factors for first-line virologic failure among children in this cohort were previously reported [16]. Of those who switched to second-line ART due to virologic failure, 70 (87.5%) failed NNRTI-based first-line ART, eight (10%) failed PI-based first-line ART and two (2.5%) failed non-standard dual therapy. Clinical and demographic characteristics for this cohort are located in Table 1. The median time to ART failure in this cohort was 95 weeks (interquartile rage (IQR) 65–147 weeks). There was no difference in time to failure between those who failed NNRTI-based first-line and PI-based first-line (median 93 vs. 107 weeks, respectively; p = 0.36). Children who failed a PI-based first-line regimen were younger (p = 0.0006), had higher absolute CD4 counts (p = 0.005) but not percentages, and had a greater increase in absolute CD4 from baseline prior to the time of regimen change compared to those who failed NNRTI-based first-line (p = 0.012).

**Table 1:** Baseline Clinical and Demographics Characteristics of A Cohort of HIV-Infected Children Failing 1^st^ Line ART in Durban, South Africa Stratified by Initial Treatment Regimen and Presence of Resistance Testing.

Covariate	NNRTI regimen	PI regimen	p-value	Resistance testing available	Resistance testing not done	p-value
	N = 70	N = 8		N = 41	N = 39	
**Median Age at initiation (years)**	6.8 [3.7–9.5]	1.2 [1.0–2.1]	0.0006	5.8 [2.6–8.0]	6.6 [2.5–9.7]	0.51
**Females**	31 (44%)	2 (25%)	0.3	17 (41%)	18 (46%)	0.68
**Cd4 Median (cells/µL)**	421 [273–663]	883 [450–1238]	0.005	441 [303–735]	434 [280–630]	0.59
**CD4 percent**	18.1 [12]–[25]	23 [16]–[28]	0.36	19 [13]–[25]	19 [13]–[26]	0.51
**Change in CD4 from baseline**	189 [34–392]	685 [70–881]	0.012	190 [43–417]	180 [54–485]	0.49
**Change in CD4% from baseline**	7.7 [1.8–14.1]	8.9 [4.3–24.6]	0.42	7.2 [2]–[14]	10 [2.5–17]	0.31
**Weeks on ART**	93 [66–155]	107 [64–113]	0.36	87 [63–132]	95 [67–168]	0.36
**Viral load at change**	10,200 [3,760–51,000]	49,000 [6,400–84,000]	0.7	16,000 [6,300–55,000]	6800 [1,900–75,300]	0.19
**Resistance Testing**	34 (49%)	6 (75%)	0.16			
**NNRTI based 1^st^ line**				34 (85%)	36 (95%)	0.16

### Response to second-line ART

Six months after regimen change, virologic suppression was 80% (53 of 66) in the PI-based second-line group and 25% (2 of 8) in the NNRTI-based second-line group (p = 0.009). We performed univariate logistic regression to determine correlates of viral suppression six months after changing to second-line ART (Table 2). We found that females (p = 0.025) and children taking NNRTI-based second-line therapy (p = 0.0093) had significantly worse viral suppression rates at six months. Using multivariate logistic regression, controlling for age, sex, first-line treatment regimen and resistance testing (Table 3), we found that children taking PI-based second-line regimen were more likely to have viral suppression six months after changing ART regimen compared to those on an NNRTI-based second-line [95% CI 2.7–232.7; p = 0.015]. In addition, females were more likely to experience virologic failure six months after initiating second-line ART [95% CI 1.4–25.3; p = 0.005]. Age (p = 0.56) and resistance testing (p = 0.22) were not significantly associated with viral suppression rate, as indicated in Table 3.

**Table 2:** Univariate Analysis: Predictors of Six Month Viral Suppression After Change to Second-Line ART in a Cohort of HIV-Infected Children Failing 1^st^ Line ART in Durban, South Africa.

	Odds Ratio [95% CI]	P value
**Age at initiation (years)**	0.99 [0.87–1.14]	0.9453
**Females**	3.60 [1.17–11.06]	0.0253
**Cd4 Median (cells/µL)**	1.00 [0.99–1.00]	0.6550
**CD4 percent**	0.96 [0.90–1.03]	0.2391
**Change in CD4 from baseline**	1.00 [0.99–1.00]	0.5965
**Change in CD4% from baseline**	0.96 [0.91–1.02]	0.1932
**Weeks on ART**	1.00 [0.99–1.01]	0.8033
**NNRTI 1^st^ line**	0.10 [0.02–0.56]	0.0093
**Log Viral load at change**	0.96 [0.72–1.28]	0.7652
**Resistance testing**	2.48 [0.82–7.55]	0.1096

**Table 3:** Multivariate Analysis: Predictors of Six Month Viral Suppression After Change to Second-Line ART in a Cohort of HIV-Infected Children Failing 1^st^ line ART in Durban, South Africa.

	Odds Ratio [95% CI]	P value
**Age at initiation (years)**	1.05 [0.89–1.25]	0.5564
**Females**	5.99 [1.42–25.34]	0.0150
**NNRTI 1^st^ line**	0.04 [0.004–0.37]	0.0047
**Resistance testing**	2.17 [0.63–7.62]	0.2196

### Resistance testing

In this cohort, 41 consecutive children received antiretroviral resistance testing prior to changing to second-line ART under a separate research protocol for children failing first-line ART. Among children who required a change to second-line ART, there was no significant difference in baseline treatment regimen between those who received testing or those who did not; 34 (83%) received NNRTI-based first-line treatment and six (15%) received PI-based first-line treatment (p = 0.16). Resistance testing was performed on one child receiving non-standard dual therapy. Additionally, there was no difference in age, sex, CD4 count at time of regimen change, viral load prior to regimen change, or time from ART initiation to regimen change between those who received resistance testing and those who did not (Table 1). The rate of viral suppression 6 months after regimen change was similar among those with resistance testing (61%) and without resistance testing (79%) (p = 0.11). Of the children who failed to achieve viral suppression six months after switching to NNRTI-based second-line therapy, all (100%) had NNRTI resistance mutations even though they did not receive NNRTI first-line therapy. Of the two (25%) children who did achieve viral suppression on NNRTI-based second-line ART, both had only PI and NRTI class mutations. Only one (4%) child who failed NNRTI-based first-line ART had minor PI mutations while four (67%) children who failed first-line PI regimens had major NNRTI mutations.

## Discussion

Sub-Saharan Africa continues to carry the burden of new HIV infections. More than 70% of all new HIV infections occur in the region [1], and fewer than half of all HIV-infected pregnant women in sub-Saharan Africa receive an intervention to prevent HIV transmission to their children [1]. However, there has been a dramatic increase in the availability of antiretroviral therapy for children in sub-Saharan Africa since 2005 [1]. This increased access to first-line ART in children means that, over time, more children will fail first-line regimens and require second-line ART, particularly in poorly monitored or rural clinics [17], [18]. Unfortunately, in many resource-limited settings, access to second-line pediatric ART is challenging and options are very limited [19].

In South Africa, children less than 3 years of age weighing less than 10 Kg initiate a PI-based ART regimen containing lopinavir/ritonavir due to exposure to single dose nevirapine (NVP) through PMTCT programs [15]. IMPAACT P1060 demonstrated lopinavir/ritonavir's superiority in efficacy and safety to NVP as first-line treatment in PMTCT exposed and unexposed children [12], [20]. Although lopinavir/ritonavir has a relatively high genetic barrier to the development of resistance [21], [22], its effectiveness is limited by poor palatability, metabolic complications [23], and drug-drug interactions [24], [25]. In a high-burden tuberculosis area, the interaction between lopinavir/ritonavir and rifampicin can lead to sub-therapeutic drug levels and virologic failure [16], [26]. In resource-limited settings, when these children fail first-line PI-based ART, they are limited to NNRTI-based second-line regimens.

We have shown that, in our cohort, NNRTI-based second-line ART was not an optimal choice since 75% of children failed this regimen. It is possible that archived NNRTI resistance due to single dose NVP exposure led to this failure; however, since this was a retrospective study, we were unable to accurately assess exposure to single-dose NVP. Studies have found that up to 19% of women exposed to single dose NVP carry resistant mutations [27]. These mutations could be transmitted to their children through failed PMTCT or transmitted to subsequent children. HIVNET 012 indicated that 46% of children who fail PMTCT carry NVP resistance mutations [27], indicating high-level NNRTI resistance occurs in areas using single-dose NVP-based PMTCT strategies. Other studies have demonstrated even higher levels of transmitted resistance in failed PMTCT regimens [13].

The Nevirapine Resistance Study (NEVEREST) evaluated whether prior exposure to single dose nevirapine would affect outcomes in children who initially achieved viral suppression with a lopinavir/ritonavir-containing first-line regimen. The study indicated that children who initially achieved viral suppression but switched to a nevirapine-containing first-line regimen had 10 times higher risk of developing viremia >1000 copies/ml compared to those that remained on lopinavir/ritonavir-containing first-line ART [11]. In anticipation of increasing pediatric first-line PI failures, data from PENPACT-1 suggest that children failing PI-based first-line ART could delay switching to second-line ART given the low risk for selecting additional NRTI and PI mutations [28]. Given the limited second-line options for these children, delaying regimen change would seem reasonable. However, there remains an urgent need for increased access to more pediatric formulations of ART in resource-limited settings. Otherwise, children failing first-line PI regimens will have extremely limited second-line options.

In resource-limited settings, HIV resistance testing is not widely available due to expense. Currently, South Africa has a low prevalence (<5%) of transmitted PI resistance in children [5]. However, transmitted NNRTI resistance in children is classified as intermediate (5–15%) due to single-dose NVP exposure through PMTCT [5]. This severely limits available ART regimens for children. Given the intermediate level of transmitted NNRTI resistance and evidence of inferior viral suppression when used as first or second-line ART, this currently leaves limited options for children in South Africa after they fail PI-based first- or second-line therapy. Although Darunavir and Tipranavir have shown promising efficacy with limited toxicity in treatment-experienced children with significant PI resistance mutations, unfortunately these agents are not widely accessible to children in South Africa or other resource-limited settings at this time [19], [29], [30].

In this setting, females were significantly less likely to reach viral suppression after six months of second-line ART compared to males. Gender differences in mortality [31], [32], baseline CD4 [32], [33] and baseline viral load [32], [33], [34] have been seen in African pediatric HIV cohorts. Behavioral, socioeconomic, genetic, and hormonal risk factors could contribute to the differential responses to infectious diseases between males and females.

This study has several limitations. First, it was a retrospective study; therefore, we relied on previously captured data. We were unable to reliably assess adherence or identify children who were exposed to single-dose NVP through PMTCT. This could limit the generalizability of this study to other resource-limited settings. In addition, there were a relatively low number of children who failed first-line PI-based therapy, thereby limiting the power of the study.

### Conclusion

In settings of high NNRTI use for prevention of perinatal transmission of HIV, the use of NNRTI-based second-line ART after failure of a boosted PI-containing first-line regimen may result in poor virologic outcomes. Since children who develop rebound viremia on boosted PI first-line regimens are slow to develop major PI resistance mutations, these children may still achieve viral suppression with improved adherence. Improvement of current boosted PI pediatric formulations, such as co-formulated sprinkles that could improve adherence and palatability, are necessary. Newer agents or classes of ART with improved toxicity profiles, palatability and decreased drug interactions are needed in resource-limited settings.
